# COVID-19 Vaccination Hesitancy among Youths in Soweto, South Africa

**DOI:** 10.3390/vaccines11050960

**Published:** 2023-05-09

**Authors:** Jelioth Muthoni, Kennedy Otwombe, Dineo Thaele, Isaac Choge, Bent Steenberg, Clare Cutland, Shabir A. Madhi, Andile Sokani, Nellie Myburgh

**Affiliations:** 1 African Leadership in Vaccinology Expertise, Faculty of Health Sciences, University of Witwatersrand, Johannesburg 2193, South Africa; 2 Perinatal HIV Research Unit, Faculty of Health Sciences, University of the Witwatersrand, Johannesburg 2193, South Africa; 3 School of Public Health, Faculty of Health Sciences, University of the Witwatersrand, Johannesburg 2193, South Africa; 4 South African Medical Research Council Vaccines and Infectious Diseases Analytics Research Unit, Faculty of Health Sciences, University of the Witwatersrand, Johannesburg 2193, South Africanellie.myburgh@wits-vida.org (N.M.)

**Keywords:** South Africa, COVID-19, public health, Soweto, vaccination hesitancy, youths

## Abstract

In combatting COronaVIrus Disease 2019 (COVID-19), immunization is the most prominent strategy. However, vaccination hesitancy—meaning delays in accepting or denying inoculation regardless of availability—has been identified as an essential threat to global health. Attitudes and perceptions play a pivotal role in vaccine acceptability. Meanwhile, uptake in South Africa’s rollout has been particularly disappointing among youths. For that reason, we explored attitudes and perceptions of COVID-19 in 380 youths in Soweto and Thembelihle, South Africa, between April and June 2022. A staggering hesitancy rate of 79.2 percent was recorded (301/380). We found negative attitudes and confounded perceptions of COVID-19 to be fueled by medical mistrust and misinformation, with online channels as the main sources of non- and counterfactual claims stemming mostly from unregulated social media popular with youths. Understanding its underpinnings—and enhancing means of curbing vaccine hesitancy—will be paramount in boosting uptake in South Africa’s immunization program, particularly among youths.

## 1. Introduction

Coronavirus disease 2019 (COVID-19) is a contagious disease caused by Severe Acute Respiratory coronavirus (SARS-CoV-2 virus) [1,2]. COVID-19 is the first disease in history to cause a concurrent pandemic on all continents, and although largely controlled, it is currently ongoing with high morbidity and mortality rates. According to the World Health Organization (WHO), as of March 2023, there were 760 million confirmed cases of COVID-19 and 6.8 million death worldwide [3]. Specifically in Africa, from March 2020 to December 2022, 9.5 million cases were confirmed, with 174,204 deaths [3]. In South Africa, from January 2020 to March 2023, there were 4 million cases and 102,598 deaths [4]; these data are being updated regularly [3].

Vaccines have played a significant role in reducing disease severity globally [5]. Immunization is the most prominent strategy in the global fight against the virus [2]. The first effectiveness study was based on Israeli national surveillance data, which showed that from the first four months of vaccination, two doses of the Pfizer-COVID-19 vaccine reduced both symptomatic and asymptomatic infections, hospitalizations, severe disease, and mortality [2]. The Moderna COVID-19 vaccine had a 94.1 percent efficacy in preventing illness [6].

In 1796, Edward Jenner introduced vaccines as he inoculated a 13-year-old boy with the vaccinia virus (cowpox), who subsequently developed immunity to smallpox. This led to the first development of the smallpox vaccine in 1798 [7]. Today, vaccines have proven to be one of the most dependable and cost-effective public health interventions available, saving lives every year [5,8]. Over the 18th and 19th centuries, systemic implementation of mass smallpox immunization culminated in global eradication in 1979 [7]. Over time, a wide array of vaccines has been developed using different technologies, such as protein subunit, nucleic acid, viral vector, and whole virus [9]. Recently, researchers have embraced these four technologies in the fight against the COVID-19 pandemic [9].

A national COVID-19 immunization program was implemented in South Africa in February 2021 with the stated aim of inoculating 66 percent of the population (40 million) [10]. Today, only about 33 percent (20 percent) have finished their vaccination. An initial phase focused on healthcare workers, while a second phase prioritized essential workers, people over the age of 60, and adults with comorbidities. The third phase comprised the remaining population [11]. It is generally understood that to reduce the number of hospitalizations and mortality rates, vaccination coverage must be increased to sufficiently high levels. However, vaccine hesitancy and denialism, not only in South Africa but globally, is a major stumbling block [5,12]. Given the coronavirus epidemic and promises for quick development and implementation of a vaccine, the spread of anti-vaccination misinformation through social media has given it additional urgency. The Strategic Advisory Group of Experts in Immunization (SAGE), a carefully chosen team of WHO employees, looked at the escalating patterns related to the vaccination process and came up with the concept of vaccine hesitancy. In relation to vaccine hesitancy, the WHO has identified three criteria (the “three C’s”) that are crucial: (1) Complacency refers to the perception of vaccine risks, side effects, and value. (2) By convenience, we mean the vaccine’s price, accessibility, and availability, as well as how many elements are connected to it. (3) Confidence is related to confidence in the government and the industries that produce the vaccinations, as well as the efficacy and safety of vaccines [13]. Vaccine hesitancy studies have been undertaken among healthcare workers in countries such as Jordan, Uganda, and South Africa [5,11,14]. However, no research has been carried out in Soweto and Thembelihle among young people (aged 18–35). Since only a few young people have comorbidities that would expose them to severe COVID-19, the majority present with asymptomatic to mild symptoms [15]. As a result, when they are unvaccinated, they pose a considerable risk to their communities [16]. Moreover, young adults have a higher potential to spread COVID-19 because of their higher mobility and propensity to socialize and largely ignore public health guidelines [17]. Despite low levels of vaccine acceptancy [18], only a little research has been conducted in South Africa and none in Soweto. However, attitudes and perceptions of COVID-19 have been reported in the Democratic Republic of Congo and Uganda among healthcare workers to play a pivotal role in vaccine acceptability [18,19]. A study performed in Ethiopia among healthcare workers in May 2021 recorded negative attitudes and poor perceptions towards COVID-19 vaccination were contributing factors to vaccine hesitancy [20]. Moreover, the WHO Strategic Advisory Group of Experts on Immunization (SAGE) has emphasized specific attitudes and perceptions are contributing factors to vaccine hesitancy [13]. Hence, we aim to bridge that gap by scrutinizing attitudes and perceptions among young people in South Africa.

## 2. Methodology

### 2.1. Study Design and Setting

This cross-sectional study recruited participants in the Soweto and Thembelihle Health Demographic Surveillance Site (SaT HDSS), which consists of nine township clusters. Two clusters (Meadowlands and Thembelihle) were selected from the nine clusters as these are home to the highest numbers of youths. Here, residential units with members in the 18–35 age range were identified. Unique identities were given to the identified households for the purpose of a random sampling of informants. Residential units with an even number were chosen to ensure randomization. An even number is every number that can be divided by two. Voluntary participation was requested of all informants. The number of participants was increased until each cluster had a sample size of 190.

### 2.2. Description of Variables

A Likert-scale format was used, ranging from ‘strongly agree’ to ‘strongly disagree’ for six variables. The six variables measured attitudes and perceptions of COVID-19. The following scoring was used for positive statements: 4 = strongly agree, 3 = agree, 2 = disagree, and 1 = strongly disagree. The reverse was applied for negative statements, i.e., 1 = strongly agree, 2 = agree, 3 = disagree, and 4 = strongly disagree. Reliability tests were not known from other studies. However, the variables were adopted from previous WHO [21] and South African studies [11].

#### 2.2.1. Dependent Variables

Vaccine hesitancy (VH) was dependent. VH was defined with the question, ‘COVID-19 vaccination is not important’. The variable had two outcome variables: hesitant and non-hesitant. Informants who responded as ‘strongly agree’ and ‘agree’ were classified as hesitant and coded 1—while those who responded as ‘disagree’ and ‘strongly disagree’ were classified as non-hesitant and coded 0. We found negative attitudes and perceptions of COVID-19 to be clearly associated with higher levels of vaccine hesitancy [18].

#### 2.2.2. Independent Variables

Attitudes towards COVID-19 were measured by a 3-item scale (α = 0.46). Examples of items included: ’If a teacher is COVID-19 positive, they should be allowed to continue teaching’; ‘I prefer breaking physical contact with people who are COVID-19 positive’; and ‘It is better to develop immunity by getting sick rather than by getting a vaccine’.

Perceptions of the COVID-19 vaccine were measured by a 3-item scale (α = 0.24). Examples of items included: ‘I believe that the COVID-19 vaccine can stop me from being infected’; ‘Quarantining people with COVID-19 is the best way to prevent the disease’; and ‘COVID-19 vaccine has serious side-effects’.

### 2.3. Sample Size Determination

An estimate of vaccination reluctance in the study population was used to calculate the sample size. The right sample size was chosen using Calculator.net. Considering the large number of youths aged 18–35 in the catchment area at the time [6,15,22], we used a 95 percent confidence interval with a precision of ±5 percent and a 50 percent response distribution since there was no published prevalence of COVID-19 vaccine hesitancy in Soweto. This resulted in a sample size of 380 [23].

### 2.4. Ethics Clearance

The study was approved by the University of the Witwatersrand’s Human Research Ethics Committee on 28 February 2022, under certificate number H22/01/16.

### 2.5. Inclusion and Exclusion Criteria

Youths of 18–35 years were included in the study, while those below and above the age limit were excluded.

### 2.6. Targeting Participants

The researcher and research assistant paid a courtesy visit to the gatekeepers of the two cluster groups, namely, Meadowlands and Thembelihle. SaT HDSS has a community advisory board that has several community leaders of high reputation and reliability. These are referred to as gatekeepers. The gatekeepers approached in the study are the same who are involved in the SaT HDSS. The gatekeepers guided us to the homes that had our participants of interest.

### 2.7. Consenting

After identifying a study participant who met the inclusion criteria, the youths were explained what the study entailed. In a language they easily understood. An information sheet was administered explaining the study. Thereafter the participants were asked if they were willing to participate in the study without coercion or if they had any questions. When the participant accepted to participate in the study, a hard copy informed consent form was administered. Throughout the process and even in data collection, COVID-19 protocols were upheld; whereby the social distance was maintained, face masks were on, and hand sanitization was performed before administering and signing the consent form. Data collection commenced on 23 April 2022 and came to a closure on 21 June 2022.

### 2.8. Data Management

All field data were collected using tablets with REDCap software [24]. Data were tabulated using Microsoft Excel software and subsequently analyzed with STATA v13 software [25]. All electronic devices were password protected. Data were stratified on VH, which was defined as the delay in acceptance or refusal of vaccines despite availability. No scales were used.

### 2.9. Statistical Methods

Descriptive statistics were determined by age, sex, education, marital status, ethnic group, religion, and source of COVID-19 awareness and information. The distribution by VH of categorical predictive variables was compared using chi-square tests.

## 3. Results

### 3.1. Demographic Social Characteristics of the Study Sample

Data were collected from SaT HDSS (50% from each). The demographic characteristics of the population selected are shown in Table 1 below. As highlighted, the following was observed: the group aged 18–24 was predominant (62%); women were the majority (60%); 97% were ethnic Blacks; and most were unmarried (96%). Most participants were Christians (73%), followed by African traditional beliefs (20%).

#### Social Demographics and Vaccine Hesitancy

Table 1 presents socioeconomic demographics based on levels of COVID-19 VH. The following fields were highlighted as having higher VH: age 18–24 years (59%); woman (62%); secondary level of education (86%); Black ethnic group (96%); unmarried status (96%); religion (37% Christian protestant); and informants who received information from the Internet (50%). Moreover, the Internet was the most significant source of information with 49%, followed by TV with 24%.

### 3.2. Attitudes towards COVID-19 Disease and Vaccines

The first objective was attitudes toward COVID-19 among youths in Soweto. The objective used the following variables: ‘If a teacher is COVID-19 positive, they should be allowed to continue teaching’; ‘I prefer to break physical contact with people living with COVID-19′; ‘It is better to develop immunity by getting sick rather than getting the vaccine.’

#### 3.2.1. If a Teacher Is COVID-19 Positive, They Should Be Allowed to Continue Teaching

**Figure 1:**
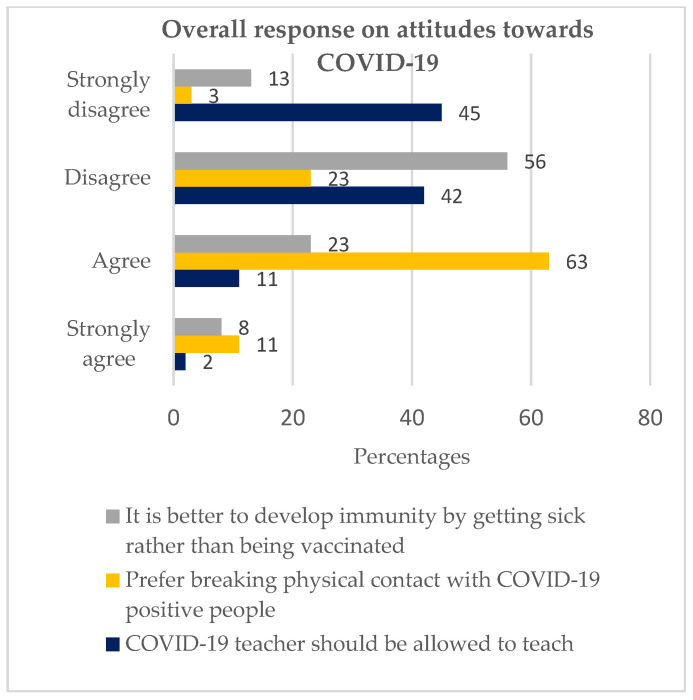
Overall response on attitudes towards COVID-19 (Chi-square test performed. Moreover, the responses, strongly agree and agree, were combined into ‘agree’ while strongly disagree and disagree were combined into ‘disagree’).

This statement was found to have substantial statistical significance (*p* value = 0.009). 87% of our informants disagreed with the statement, whereas 13% agreed with it, as illustrated in Figure 1.

Of the 87% (Figure 1) who disagreed (both ‘disagreed’ and ‘strongly disagreed’), 81% were hesitant (Table 2), while of the 13% (Figure 1) who agreed (both ‘strongly agreed’ and ‘agree’), 67% were hesitant, as depicted in Table 2.

#### 3.2.2. I Prefer to Break Physical Contact with People Living with COVID-19

The statement had a statistical significance of *p*-value = 0.045. Seventy-four percent agreed with the statement, while 26% disagreed, as illustrated in Figure 1. Of the 74% (Figure 1) who agreed with the statement, 81% (Table 2) were hesitant, while of the 26% (Figure 1) who disagreed, 74% were hesitant, as depicted in Table 2.

#### 3.2.3. It Is Better to Develop Immunity by Getting Sick Rather than by Getting a Vaccine

The statement was shown to have a high statistical significance of *p*-values = 0.000. 69% disagreed with the statement, while 31% agreed, as illustrated in (Figure 1). Of the 69% who disagreed, 86% were hesitant, while of the 31% who agreed, 64% were hesitant, as depicted in Table 2.

### 3.3. Perceptions of COVID-19

The next objective was to investigate perceptions of COVID-19 among youths in Soweto. The objective used the following variables: ‘I believe that the COVID-19 vaccine can stop me from being infected’; ‘Quarantining people with COVID-19 is the best way to prevent the disease’; ‘COVID-19 vaccine has serious side-effects’.

#### 3.3.1. I Believe That the COVID-19 Vaccine Can Stop Me from Being Infected

**Figure 2:**
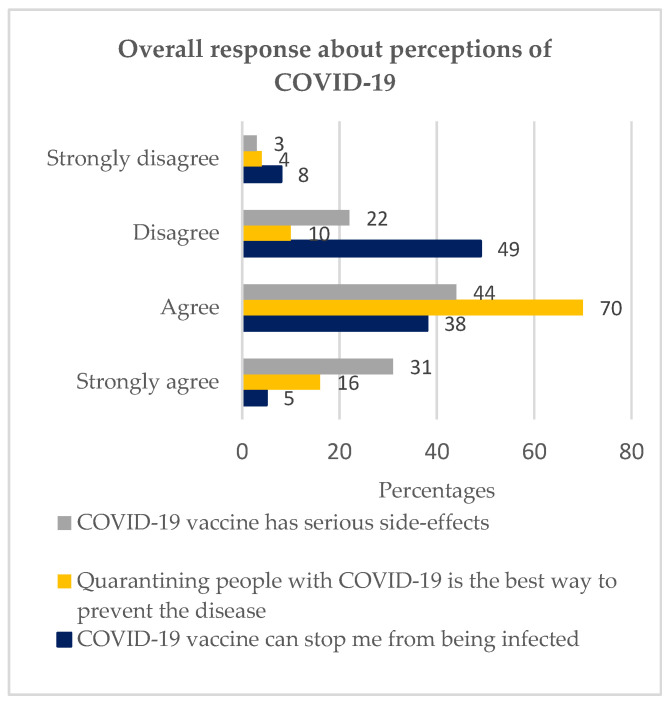
Overall response about perceptions of COVID-19 (Chi-square test performed. Moreover, the responses, strongly agree and agree, were combined into ‘agree’ while strongly disagree and disagree were combined into ‘disagree’.).

57% disagreed with the statement, while 43% agreed, as illustrated in Figure 2. A statistical significance of 0.000 was recorded.

Of the 57% who disagreed (Figure 2), 69% were hesitant (Table 2), while of the 43% who agreed (Figure 2), 92% were hesitant, as depicted in Table 2.

#### 3.3.2. Quarantining People with COVID-19 Is the Best Way to Prevent the Disease

86% agreed, while 14% disagreed. A statistical significance of 0.000 was captured. Of the 86% who agreed (Figure 2), 83% were hesitant (Table 2), while of the 14% who disagreed (Figure 2), 55% were hesitant, as depicted in Table 2.

#### 3.3.3. COVID-19 Vaccine Has Serious Side-Effects

75% agreed, while 25% disagreed, as illustrated in Figure 2. A statistical significance of *p* = 0.000 was captured. Of the 75% who agreed, 83% were hesitant, while of the 25% who disagreed, 68% were hesitant, as depicted in Table 2.

## 4. Discussion

According to the WHO, vaccine hesitancy is one of the primary threats to global health [11]. However, little is known about the causes and nature of VH in South Africa. Whereas negative attitudes and perceptions have been clearly associated with VH [26], there are little data on youths (18–35 years). Our research elucidated a level of COVID-19 VH of 79.2%. Moreover, 20.8% does not mean they are fully vaccinated. This is attributable to ubiquitous negative attitudes and perceptions of COVID-19, with the Internet (and most social media platforms) as the main sources of dis- and misinformation.

Internet use was the most prominent source of information (49.5%), which concurs with other studies [27,28]—most likely because these all focused on youths. There are higher levels of peer pressure among single people compared to people living together/cohabiting/married, which may explain their higher level of VH. In line with the level of education, those with some secondary levels of education are likely to have some information about COVID-19, but not comprehensively enough to guide them towards a concrete decision about whether to inoculate or not. At the same time, those with primary/no formal education have little grounds for reasoning against COVID-19 immunization. This is consistent with a study on teachers working in preschools and in higher education, which found that the less educated were less hesitant than those with higher education [29]. Moreover, people who obtain information from the Internet—specifically from unregulated social media platforms commonly used by young people across the globe—demonstrate a higher chance of being ensnared to misinformation and swayed into VH [22].

This next section deliberates important elements of attitudes towards COVID-19, which include whether a teacher should be allowed to teach while positive, preference for breaking physical contact with COVID-19-positive people, and preference for developing immunity by getting sick rather than getting a vaccine.

87% disagreed with the statement, which means they would prefer teachers to follow. COVID-19 prevention measures, including self-isolation. This infers that informants were aware that one could transmit the virus while in close contact. Thus, only 13% agreed with the statement and so likely believed that ‘COVID-19 is not a real’ or was in some state of fatalism.

Despite the 87% majority who were cognizant that COVID-19 was real via their preference for social distancing, a surprisingly high rate of VH was captured among them, namely 81%. Perhaps this is down to thinking along the lines of ‘if the COVID-19-positive teacher is in self-isolation, there is no need to immunize’ since they are now distanced. This is coherent with a study on community health workers; the majority preferred distancing from COVID-19-positive individuals [30]. This may be attributed to a similar age group (20–30 years) and a similar population (Soweto). Moreover, in a study on HIV among Gabolese and Malians living in Gabon, informants expressed high rates of fatalism, despite acknowledging that HIV was real, as we also found here with COVID-19.

74% agreed to break physical contact with a COVID-19-positive individual. This suggests that our informants were indeed aware of COVID-19′s existence. However, 26% disagreed with the statement. Interestingly, a higher rate of VH was recorded among those who agreed (81%) compared with those who did not (74%). This suggests that people who prefer breaking physical contact see little need for a vaccine—having chosen this ‘safer’ and easier way. Again, this is in line with similar studies [30]. Moreover, some have pointed to the fact that COVID-19 disease affects mostly the elderly as an explanation as to why young people are less inclined to immunize [15]. In short, they see no need to break physical contact out of a perception of being safe and of possessing strong immunity. Informants who disagree with the statement are likely to believe that COVID-19 is not real or to exist in some state of fatalism. Again, another South African study—but this time on HIV—had similar findings [31]. Despite of differences in study design, this correlation could be attributed to sentiments of hopelessness that progress into fatalism.

Sixty-nine percent, or well over half of our informants, disagreed with the statement through a preference for getting sick rather than inoculating. However, a substantial proportion (31%) preferred getting sick rather than immunized. Of the 69% who disagreed, 86% were hesitant. Reasons underlying such elevated hesitancy rates could be associated with sources of information, as 49% of informants recounted using the Internet. Internet users had the highest proportion in the hesitant group (50%). Recent research has suggested that the Internet is a primary source of misinformation and ‘infodemics’, which can translate into VH [22,32]. Furthermore, a meta-analysis survey, which investigated the acceptability of COVID-19 vaccination in South Africa from February 2020 to March 2021, recorded that 40% agreed with the statement that infection-acquired immunity is better than vaccine-acquired immunity [11]—slightly higher than here. This is attributable to the difference in study populations, as this present study was the first to be undertaken in this SaT HDSS. Moreover, the time periods for data collection were dissimilar (here, April to June 2021). Furthermore, our study went one step further by analyzing VH rates in groups that either agreed or disagreed.

In the next section, we will address elements of perceptions of COVID-19 related to vaccination hesitancy. This includes: ‘COVID-19 vaccine can stop one from being infected,’ ‘Quarantine is the best way to prevent COVID-19 disease,’ and ‘COVID-19 vaccine has serious side-effects’.

Our findings had a tentative 50/50% response that disagreed (57%) or agreed (43%) with the statement ‘COVID-19 vaccine can stop one from being infected’. This reveals the degree of uncertainty experienced by our informants, suggestive of a lack of knowledge and information. However, a fair share (57%) was knowledgeable about the role of COVID-19 vaccines in that they protect against severe disease, hospitalization, and mortality rather than infection [33]. Of the 57% who disagreed, 69% were hesitant. This could be due to a lack of information or exposure to misleading (mis)information [22,34]. This explains why a higher VH rate (93%) was recorded in the group that agreed compared with those who disagreed because the group that agreed did not expect to be infected with COVID-19. However, when they were, they had high levels of medical mistrust, resulting in VH. As with our findings, similarly, a Canadian longitudinal observational study recorded a lack of trust in the COVID-19 vaccine to be prevalent among hesitant people aged 18–65 year [34].

In our findings, most of the study population (86%) considered quarantine to be the best way of preventing COVID-19. This was complemented by most of our informants having a preference for a COVID-19-positive teacher to not be allowed to teach (87%) and breaking physical contact with COVID-19-positive people (74%). In most of the informants who preferred to quarantine, a higher VH level (83%) was recorded compared to the people who disagreed with the statement. Hence, the population is inclined towards breaking physical contact with people who are positive for COVID-19 rather than being vaccinated. To informants, this was the ‘easier’ or ‘safer’ way, demonstrating a lack of concrete information. However, this approach is biased as most young people tend to present with asymptomatic to mild symptoms. As a study about patients classified based on the severity of COVID-19 depicts similar findings [15]. Hence young people form a fertile ground for being super-spreaders. Research has recorded high rates of infection in ages 20–49 compared to other groups and high rates of mortality in ages 60 and above [34]. This shows that the population is knowledgeable but possesses lacunas in this knowledge. As we have underscored, the Internet was the main source of information for informants. The Internet is inclusive of social media in which misinformation and misconceptions abound [22,34]. Here, unlike any other research, we have accentuated levels of VH in relation to responses.

Of all informants, 75% perceived vaccines to have serious side effects. Almost a quarter (25%) disagreed with the statement. Out of the 75% who agreed, 83% were hesitant. Those who disagreed had a lower hesitancy level (68%). Studies have indicated side effects to be one of the top reasons for VH [35]. For instance, a 2021 study from Vietnam reported that students mentioned COVID-19 vaccination side effects to be the main reason underpinning VH [33]. Interestingly, of the informants who disagreed, a substantial proportion were still hesitant. This could be due to knowledge not translating into behavior, as illustrated copiously in the HIV literature [36]. Finally, peer pressure is more prominent in the younger population [37].

## 5. Conclusions

This is the first study exploring VH among youths in Soweto and Thembelihle, South Africa. Here, we accentuated that informants had negative attitudes and perceptions toward COVID-19 immunization, which contributed to staggeringly high rates of VH and denialism. Clearly, as we saw, this was attributable to misinformation stemming from the Internet (and mostly unregulated social media platforms) as the primary source of non- or counterfactual claims. This suggests addressing the negative attitudes and perceptions among the youth would boost the COVID-19 vaccine uptake tremendously. Our study depicts a preference for social distancing with COVID-19-positive people. Deducing people are aware COVID-19 is real but recording high levels of VH, hence exhibiting a state of fatalism. Moreover, having a misperception of being ‘safe’ when quarantine and isolation measures are put in place yields VH. Furthermore, the perception of COVID-19 vaccines having serious side-effect that is exaggerated in society is a contributing factor to VH. Our study justifies on the urgency to address negative attitudes and perceptions among the youth in Soweto. Moreover, the study recommends a replica in other South African provinces.

### 5.1. Study Strengths

- This is the first study to highlight vaccine apprehension in Soweto among the youths.
- The quantitative study design embraced a larger population, hence highlighting the solid state of the attitudes and perceptions on the COVID-19 vaccine.
- The study design objectively and accurately brought out the levels of attitudes and perceptions of COVID-19 vaccines that correlate with vaccine hesitancy.
- The study uniquely depicted the different levels of COVID-19 vaccine hesitancy based on the responses received.

### 5.2. Study Limitations

- The quantitative study design was limited to giving reasons behind negative attitudes and poor perceptions,
- The study did not capture the vaccination status of the participants. However, most youths were obliged to vaccinate due to mandatory vaccination policy in schools and corporate environments [30].

## Figures and Tables

**Table 1:** Distribution of responses on social demographics based on levels of COVID-19 VH.

Variable	Response	Non-Hesitancy *n* (%)	Hesitancy *n* (%)	Total (380) *n* (%)	*p*-Value
Age	18–24 years	55 (70)	179 (59)	234 (62)	0.099
	25–35 years	24 (30)	122 (41)	146 (38)	
Sex	Male	37 (47)	114 (38)	151 (40)	0.147
	Female	42 (53)	187 (62)	229 (60)	
Education	Primary or None	0 (0)	3 (1)	3 (1)	0.385
	Secondary	65 (82)	259 (86)	324 (85)	
	Tertiary	14 (18)	39 (13)	53 (14)	
Marital status	Not in union/not married/ single	77 (98)	288 (96)	365 (96)	0.468
	In union/married	2 (2)	13 (4)	15 (4)	
Ethnic group	Black	79 (100)	290 (96)	369 (97)	0.226
	Colored	0 (0)	2 (1)	2 (1)	
	Non-South Africans	0 (0)	9 (3)	9 (2)	
Religion	Roman Catholic church	2 (3)	19(6)	21 (6)	0.531
	Evangelical Charismatic	8 (10)	36 (12)	44 (11)	
	African Independent Church	20 (25)	53 (18)	73 (19)	
	Christian protestant	27 (34)	112 (37)	139 (37)	
	Muslim	1 (1)	7 (2)	8 (2)	
	African tradition ^1^	18 (23)	57 (19)	75 (20)	
	Other	3 (4)	17 (6)	20 (5)	
Source of information	Family members	17 (22)	47 (16)	64 (17)	0.558
	Friends	3 (4)	6 (2)	9 (2)	
	Healthcare workers	1(1)	11 (4)	12 (3)	
	Internet	34 (44)	150 (51)	184 (50)	
	Radio/School	3 (4)	10 (3)	13 (4)	
	TV	19 (25)	71 (24)	90 (24)	
Level of significance is at *p*-value less than or equal to 0.05					

^1^ African tradition is a type of religion in which past and present elders play a significant role. People maintain a spiritual connection with their ancestors through rituals. The tradition has spiritual leaders called *sangomas* that are responsible for spiritual and physical healing, as well as counseling about the future.

**Table 2:** COVID-19 hesitancy levels were based on the informants’ responses to the statements that addressed both the attitudes and perceptions.

	“If a Teacher Is COVID-19 Positive, They Should Be Allowed to Continue Teaching.”			
Responses	Non-hesitant	Summary	Hesitant	Summary
	*n* (%)	*n* (%)	*n* (%)	*n* (%)
Strongly agree	2 (4)	17 (33)	6 (12)	34 (67)
Agree	15 (29)		28 (55)	
Disagree	39 (12)	62 (19)	122 (37)	267 (81)
Strongly disagree	23 (7)		145 (44)	
	“I prefer to break physical contact with people living with COVID-19.”			
Strongly agree	2 (1)	53 (19)	39 (14)	227 (81)
Agree	51 (8)		188 (67)	
Disagree	23 (64)	26 (26)	64 (64)	74 (74)
Strongly disagree	3 (10)		10 (10)	
	“It is better to develop immunity by getting sick rather than getting the vaccine.”			
Strongly agree	7 (6)	42 (36)	24 (21)	75 (64)
Agree	35 (30)		51 (44)	
Disagree	34 (13)	37 (14)	180 (68)	226 (86)
Strongly disagree	3 (1)		46 (18)	
	“I believe that the COVID-19 vaccine can stop me from being infected.”			
Strongly agree	2 (1)	13 (8)	19 (12)	151 (92)
Agree	11 (7)		132 (80)	
Disagree	53 (25)	66 (31)	132 (61)	150 (69)
Strongly disagree	13 (6)		18 (8)	
	“Quarantining people with COVID-19 is the best way to prevent the disease.”			
Strongly agree	4 (1)	55 (17)	55 (17)	272 (83)
Agree	51 (16)		217 (66)	
Disagree	17 (32)	24 (45)	21 (40)	29 (55)
Strongly disagree	7 (13)		8 (15)	
	“COVID-19 vaccine has serious side-effects.”			
Strongly agree	20 (7)	48 (17)	96 (34)	236 (83)
Agree	28 (10)		140 (49)	
Disagree	22 (23)	31 (32)	60 (63)	65 (68)
Strongly disagree	9 (9)		5 (5)	

## Data Availability

Data presented in this study are available on request from the corresponding author.
